# Risk of Recurrence of Chronic Subdural Hematomas After Surgery: A Multicenter Observational Cohort Study

**DOI:** 10.3389/fneur.2020.560269

**Published:** 2020-11-24

**Authors:** Fabio Cofano, Alessandro Pesce, Giovanni Vercelli, Marco Mammi, Armando Massara, Massimiliano Minardi, Mauro Palmieri, Giancarlo D'Andrea, Chiara Fronda, Michele Maria Lanotte, Fulvio Tartara, Francesco Zenga, Alessandro Frati, Diego Garbossa

**Affiliations:** ^1^Neurosurgery Unit, Department of Neuroscience “Rita Levi Montalcini”, University of Turin, Turin, Italy; ^2^IRCSS “Neuromed”, Pozzilli, Italy; ^3^Neurosurgery Unit, Department of Human Neuroscience, Sapienza University, Rome, Italy; ^4^Neurosurgery Unit, Ospedale Spaziani, Frosinone, Italy; ^5^Stereotactic and Functional Neurosurgery Unit, Department of Neuroscience “Rita Levi Montalcini”, University of Turin, Turin, Italy; ^6^Istituto Clinico Citta Studi Milano, Milano, Italy

**Keywords:** craniostomy, chronic subdural hematoma, corticosteroids, drain, recurrence

## Abstract

**Background:** Chronic Subdural Hematoma (CSDH) is a common condition in the elderly population. Recurrence rates after surgical evacuation range from 5 to 30%. Factors predicting recurrence remain debated and unclear.

**Objective:** To identify factors associated with increased risk of recurrence.

**Methods:** Cases of CSDHs that underwent surgical treatment between 2005 and 2018 in the Neurosurgery Units of two major Italian hospitals were reviewed. Data extracted from a prospectively maintained database included demographics, laterality, antithrombotic therapy, history of trauma, corticosteroid therapy, preoperative and postoperative symptoms, type of surgical intervention, use of surgical drain, and clinical outcomes.

**Results:** A total of 1313 patients was analyzed. The overall recurrence rate was 10.1%. The risk of recurrence was not significantly different between patients with unilateral or bilateral CSDH (10.4 vs. 8.8%, *p* = 0.39). The risk of recurrence was higher in patients that underwent surgical procedure without postoperative drainage (16.1 vs. 5.4%, *p* < 0.01). No relationship was found between recurrence rates and therapy with antithrombotic drugs (*p* = 0.97). The risk of recurrence was increasingly higher considering craniostomy, craniectomy, and craniotomy (9.3, 11.3, and 18.9%, respectively, *p* = 0.013). Lower recurrence rates following Dexamethasone therapy were recorded (*p* = 0.013).

**Conclusion:** No association was found between the risk of recurrence of CSDH after surgical evacuation and age, use of antithrombotic medication, or laterality. Burr-hole craniostomy was found to be associated with lower recurrence rates, when compared to other surgical procedures. Placement of surgical drain and Dexamethasone therapy were significantly associated with reduced risk of recurrence of CSDHs.

## Introduction

Chronic Subdural Hematoma (CSDH) is a common condition affecting the elderly population (>65 years). Its general incidence, estimated between 1.72 and 20.6 per 100,000 persons per year (1), is currently rising due to the increasing age and widespread use of antiplatelet and anticoagulant drugs (2). The appropriate treatment could be conservative or, when symptoms of compression occur, surgical. Burr-hole, twist drill, or craniotomy with or without drain placement are usually considered the surgical treatments of choice (3). Recurrence rates after surgical evacuation range from 5 to 30% (4, 5). Risk factors for recurrence are still debated and there is no universal consensus regarding the best surgical technique or optimal pre- and postoperative management (6, 7). The possible impact of recurrences after surgical treatment of a CSDH on the overall fatality rate is also extensively debated (8), although in most investigations and in the practical clinical situation of extremely old patients suffering from multiple major comorbidities, reliable information concerning the “disease specificity” profile of the fatality rate of CSDH recurrences appear to be still elusive and difficult to collect (8).

The aim of the present investigation is to identify risk factors for recurrence of bleeding, requiring a *repeated* surgical procedure, through the analysis of the clinical, radiological and surgical results of 1,313 surgical cases of CSDH, whose records, including presenting symptoms, hematoma characteristics, type of surgical treatment, corticosteroid treatment, were reviewed retrospectively.

## Methods

### Participants and Eligibility

In the present cohort study, we analyzed data of patients suffering from CSDH who underwent surgical treatment between June 2005 and August 2018 in the Neurosurgery Unit of “Città della Salute e della Scienza” of Turin and of the Hospitals of “Sapienza” University of Rome. Clinical management, including surgical and medical treatment, followed a homogenous protocol during the years of the study in the participating centers.

Data were extracted and analyzed retrospectively from our Institutional databases and included: demographic information (age and sex), side of bleeding, antiplatelet and/or anticoagulant therapy at presentation, history of trauma, pre- and/or postoperative corticosteroid therapy, preoperative and postoperative neurological symptoms, type of surgical intervention, use of surgical drain, and neurological outcomes.

### Inclusion and Exclusion Criteria

Clinical data belonging to patients who underwent surgery for the management of CSDH were collected. As previously specified, we collected a vast amount of data concerning these patients, in order to investigate the role played by different variables in determining the risk of recurrence and the clinical outcome. All the patients included were operated on for CSDH, this was the main inclusion criteria. Conversely, the absence or unavailability of radiological, clinical, surgical records, and in general, missing information about the follow-up were exclusion criteria. Exception done for all the patients whose radiological, clinical and surgical records presented missing data, the present cohort is a consecutive series.

### Data Source

Clinical information was obtained at the time of admission and at follow-up clinic evaluation by fully trained neurosurgeons of the Departments. Patients' age was recorded and analyzed as a continuous variable and was divided subsequently, for statistical purposes, into four groups: <65, 65–74, 75–84, and >85 years. Side of bleeding was categorized into “unilateral” or “bilateral” hematoma. Antithrombotic therapy was considered as a categorical variable: “none,” “antiplatelet,” “anticoagulant,” and “antiplatelet plus anticoagulant.” History of trauma was considered as a dichotomous variable (1/0 - “yes” or “no”).

Corticosteroid therapy was considered as a categorical dichotomous variable for the preoperative and the postoperative settings; dosage was not investigated, as it usually followed a standardized protocol consisting in an administration of i.m. or i.v. Dexamethasone at the dose of 4 mg twice a day. At admission, presenting symptoms were classified into four groups: “no symptoms,” “headache,” “focal deficit,” or “GCS < 9” (i.e., coma). Type of surgical intervention was categorized into: “craniostomy,” “craniectomy,” or “craniotomy” (see Treatment Protocol). Use of postoperative surgical drain was considered as a dichotomous variable (1/0 - “yes” or “no”). In general, the use of drainage, the surgical technique, the corticosteroid therapy protocol, and the radiological follow-up program was completely identical in the different centers.

### Outcome Variables

Main outcome variables were neurological outcomes and recurrence. The neurological conditions in the preoperative and postoperative period were analyzed with the Markwalder score (9) specifically conceived for the CSDH patients, in order to increase the comparability of our results. The neurological outcomes at last follow-up were furthermore classified into three groups: “improvement,” “stable,” “worsening”; this was considered as an ordinal variable on the ground of the variation of the Markwalder score between the pre and postoperative period. Neurological outcomes at last follow-up were further classified into four groups: “improvement,” “stable,” “worsening” or “stable in asymptomatic”; this was considered as a nominal categorical variable. Recurrence was defined as the necessity to repeat the surgical procedure, as determined by a consulting neurosurgeon, according to clinical symptoms (worsening neurological status compared to the first postoperative setting) and radiological findings (dimensional increase of the potentially residual hematoma compared to immediate postoperative and/or increase of midline shift). Variables associated with an increased incidence of recurrence were investigated. The mortality was recorded. In particular, we reported as CSDH related all the fatalities which had a direct or indirect relationship to CSDH, for instance, a myocardial infarction or a pulmonary embolism related the temporary interruption of antiplatelet drugs was considered to be related although involving other organs.

### Surgical Indication and Timing

Surgical indication, for patients both at their first surgery or at their surgery for a recurrent CSDH, was reserved for patients presenting 1 cm or more of midline shift on the admission brain CT scan, patients presenting neurological signs or symptoms related to the CSDH (such as cognitive or behavioral slowing, motor or sensory disturbances, seizures), or asymptomatic patients whose maximum thickness of CSDH, independently from the neurological symptoms, was more than 2 cm. Cognitive slowing, a key clinical finding among the CSDH symptoms was defined by an obvious slowing of thought and movement, when the patient is examined, this finding becomes especially clear when the patient dialogs and/or executes motor commands of the examiner.

Surgery was usually performed as early as possible, i.e., within 48 h from admission, according to the clinical presentation of the patient. The procedure was delayed when the results of coagulation studies showed alteration of routine parameters: international normalized ratio (INR) > 1.3, platelet count < 100,000/mL, activated partial thromboplastin time (aPTT) > 45 s. In the aforementioned cases, or when the anamnesis of the patient revealed the use of antiplatelet or anticoagulant drugs, surgery was delayed until after the normalization of parameters and/or the administration of antidotes or blood transfusion.

### Surgical Technique

Surgical technique and clinical management were chosen according to radiological findings and surgeon's preference. In most cases a craniostomy with a single parietal or frontal burr-hole was performed. Alternatively, a craniectomy (over 2 × 2 cm) was executed with an high-speed drill. In selected cases, when acute clots were detected on the CT scan, a craniotomy was preferred. According to the surgeons' preferences through the years, placement of subdural drain was not always performed. The main reason not to leave the drain in the subdural compartment was the evidence of a satisfactory intraoperative decompression of the brain with the absence of enough space between the cortical surface and the dura mater to lay the drainage. When placed, the drainage was removed after approximately 48 h and after a postoperative CT scan, confirming the satisfactory outcome of the procedure.

### Clinical Management

The clinical management protocol was homogeneous, identical in the different centers. Whenever possible, according to the other comorbidities (e.g., diabetes, osteoporosis, hypertension, glaucoma) preoperative and/or postoperative corticosteroid taper (lasting up to 2 weeks after the procedure) with Dexamethasone (starting at 4 mg iv bid) was administered. In case of the aforementioned comorbidities, seriously affected by corticosteroid therapy, the dosage and the resulting length of the treatments were halved. The follow-up program included a CT scan and clinical evaluation about 4 weeks after surgery. Management was then individualized according to the clinical and radiological findings after the procedure. The reinstitution of antiplatelet or anticoagulant drugs was planned together with handling cardiologists or primary care physicians. In case of recurrence, the same aforementioned principles for surgical indication and clinical management were followed.

### Statistical Analysis and Power of the Study

Descriptive statistics were reported as a median, mean, and standard deviation for continuous variables or frequency and percentage for categorical variables. Comparisons of proportions were performed with Chi^2^ test for categorical variables, Risk was stratified according to standard odds radio methods. Multivariate, Repeated Measures and Univariate ANOVA analyses were used for the ordinal and continuous variable, as much as Logistic Regression was used to predict the results concerning the endpoint variable. Statistical significance was defined with a *p*-value < 0.05. All statistical analyses were performed using SPSS Statistics software (IBM SPSS Statistics for Windows, Version 25.0; IBM Corp., Armonk, New York, USA). In regards to the intrinsically dichotomous nature of prediction/association (1/0 - relapse/no relapse) and in regards to the endpoints selected, the study presents an excellent *post-hoc* statistical estimated power (1 – β = 0.947 for α 0.05 and effect size “f” as low as = 0.3), thus providing extremely reliable conclusions.

### Compliance to Ethical Standards

The informed consents were approved by the Institutional Review Board of our Institutions both in regard to the clinical and research purposes. Before surgical procedure, all the patients gave informed written explicit consent after appropriate information. Data reported in the study have been completely anonymized. No treatment randomization has been performed. This study is perfectly consistent with Helsinki declaration of Human Rights.

## Results

### Participants and Descriptive Data

A total of 1,313 patients was analyzed (Table 1). Average age was 76.6 years (standard deviation 9.9, median 78); female to male ratio was 0.4/1 (403/910). In 75.8% of cases the CSDH was unilateral. Anticoagulant therapy was reported in 13.6% of cases (179 patients), while antiplatelet in 27.3% (358 patients). Data about past history were reported in 831 cases, with 458 patients reporting a traumatic event having occurred before the diagnosis (55.1%). A neurological deficit was recorded in 1,053 patients (80.5%), while less common presentations were headache (10.1%), history of incidental finding (7.0%), and coma (2.4%). In 1,161 patients (88.4%) surgery consisted in single burr-hole craniostomy. Craniectomy was performed in 4.7% of cases, while craniotomy in 6.9%. A drainage was placed in 537 patients (73.0%, data available for 736 cases).

**Table 1:** Demographics, management, and surgical data.

		**No. of patients/No. of patients for whom data are available (%)**	
Total no. of patients	1,313		
Mean age in years (SD)	76.6 (9.9)		
Median age in years	78		
Sex	F	403/1,313	(31.7)
	M	910/1,313	(69.3)
	F/M ratio	0.4/1	
Unilateral or bilateral	Unilateral	995/1,313	(75.8)
	Bilateral	318/1,313	(24.2)
Antithrombotic therapy	Anticoagulant	179/1,313	(13.6)
	Antiplatelet	358/1,313	(27.3)
Corticosteroid therapy	Preop	143/719	(19.9)
	Postop	294/719	(40.9)
History of trauma	Yes	458/831	(55.1)
	No	340/831	(40.9)
Surgical drain	Yes	537/736	(73.0)
	No	199/736	(27.0)
Operation	Craniostomy	1,161/1,313	(88.4)
	Craniectomy over 2 × 2 cm	62/1,313	(4.7)
	Craniotomy	90/1,313	(6.9)
Recurrence		132/1,313	(10.1)
Death		29/1,313	(2.2)
Discharge	Home	588/802	(73.3)
	To other hospital	214/802	(26.7)
Preop symptoms	None	92/1,308	(7.0)
	Headache	132/1,308	(10.1)
	Neurological deficit	1053/1,308	(80.5)
	GCS <9	31/1,308	(2.4)
Neurological outcome	Improvement	1,031/1,266	(81.4)
	Stable	124/1,266	(9.8)
	Worsening	9/1,266	(0.7)
	Stable in asymptomatic	102/1,266	(8.1)

### Outcome Data

Of all treated patients, the vast majority (73.3%) was discharged at home and then followed up in the outpatient services of our institution; 26.7% of patients were transferred to another hospital for neurological rehabilitation after the operation. At follow-up 29 patients were dead because of complications related to the CSDH realizing an overall disease specific mortality rate of 2.2%. Neurological improvement was recorded in the majority of symptomatic patients (81.4%). A total of 9.8% of patients showed stable postoperative symptoms, while 8.1% of patients were asymptomatic preoperatively and did not worsen. A neurologic worsening was recorded in 0.7% of all cases. In a total of 719 cases the details of the corticosteroid treatment were reported in the clinical records, A total of 401 patients did not undergo any corticosteroid treatment (55.8%), 195 patients underwent preoperative or postoperative corticosteroid treatment (14.9%), whereas 123 patients received pre and postoperative treatment (9.4%).

### Risk of Recurrence

Risk factors for recurrence were investigated and summarized in Table 2. The overall recurrence rate of the entire cohort was 10.1%. No association was found between patient age group (<65, 65–74, 75–84, and >85 years) and rate of recurrence (*p* = 0.93), but male sex was statistically associated with the risk of relapse (*p* = 0.011, OR 95%IC 1.080–2.550 – Table 2) The risk of recurrence was not significantly different between patients with unilateral or bilateral CSDH (10.4 vs. 8.8%, *p* = 0.39).

**Table 2:** Risk of recurrence.

		**Risk of recurrence [95% IC]**	***p*-value**
Age	<65	10.1% [6.2–16 %]	0.93
	65–74	10% [6.7–13.2 %]	
	75–84	10% [9.3–14.9 %]	
	>85	10% [6.5–13.6 %]	
Sex	Male	12.6% [11.3–12.9 %]	0.011
	Female	7.8% [5.7–11.3 %]	
Unilateral or bilateral	Unilateral	10.4% [8.7–12.5 %]	0.39
	Bilateral	8.8% [6.1–12 %]	
Surgical drain	Yes	5.4% [3.8–7.6 %]	<0.01
	No	16.1% [12–22 %]	
Operation	Craniostomy	9.3% [7.8–11.1 %]	0.013
	Craniectomy over 2 × 2 cm	11.3% [5.5–21.5 %]	
	Craniotomy	18.9% [12.1–28.1 %]	
Antithrombotic therapy	None	10.2% [8.2–12.5 %]	0.97
	Antiplatelet	9.5% [6.8–13 %]	
	Anticoagulant	10.6% [6.8–16.2 %]	
	Antiplatelet + Anticoagulant	10% [1–40 %]	
Corticosteroid therapy	None	11% [8.3–14.4 %]	0.013
	Preop	4.2% [ 0.7–20.2 %]	
	Postop	6.9% [3.6–11.1 %]	
	Preop + Postop	2.4% [0.8–6.9 %]	
Preop symptoms	None	5.4% [2.3–12.1 %]	0.20
	Headache	6.8% [3.6–12.4 %]	
	Neurological deficit	10.9% [9.2–12.9 %]	
	Cognitive impairment	9.5% [ 9.4%−30.7%] *p* = 0.034	
	GCS <9	9.7% [3.3–24.9 %]	
Neurological outcome	Improvement	5% [2–12 %]	0.22
	Stable	10% [8.5–12.9 %]	
	Worsening	11.7% [6.6–20.6 %]	
	Stable in asymptomatic	22.2% [6.3–54.7 %]	

**Figure 1:**
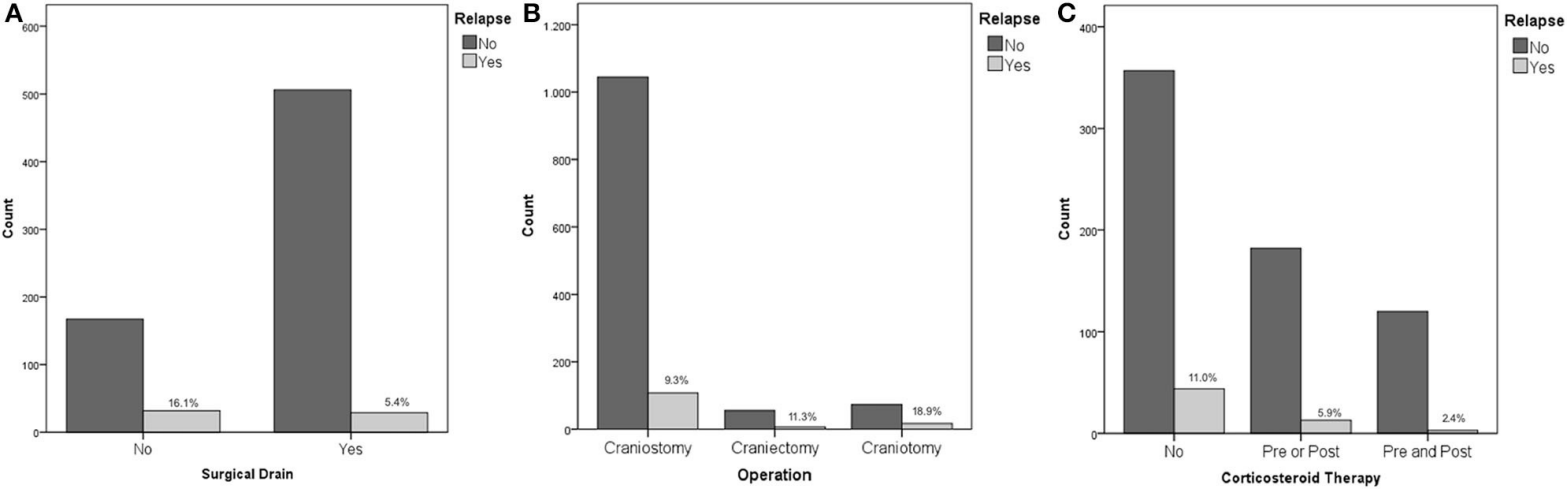
**(A)** Placement of surgical drain and risk of recurrence; **(B)** Operation type and risk of recurrence; **(C)** Corticosteroid therapy and risk of recurrence.

Recurrence rates were much higher in patients, who underwent surgical procedure without postoperative drainage, namely, positioning of surgical drainage was statistically strongly associated to a reduction of the risk of relapse (16.1 vs. 5.4%, *p* < 0.01 - Figure 1A).

No association was found between recurrence rates and therapy with antiplatelet or anticoagulant drugs, or a combination of them (*p* = 0.97). The risk of recurrence was increasingly higher considering craniostomy, craniectomy, and craniotomy (9.3, 11.3, and 18.9%, respectively, *p* = 0.013 - Figure 1B).

Considering corticosteroid therapy, lower recurrence rates following Dexamethasone therapy were recorded (*p* = 0.013). Specifically, patients that did not receive Dexamethasone at all, patients receiving therapy only before the procedure, patients that received Dexamethasone only after surgery, and patients who received corticosteroid therapy both before and after the procedure had a risk of recurrence of 11, 4.2, 6.9, and 2.4%, respectively (Figure 1C). Although pre and postoperative corticosteroid therapy was proven to be associated with a reduced relapse rate, corticosteroid administration, both in the pre and postoperative was not directly associated with a decreased fatality rate (*p* = 0.255 and *p* = 0.221 pre and post, respectively).

**Figure 2:**
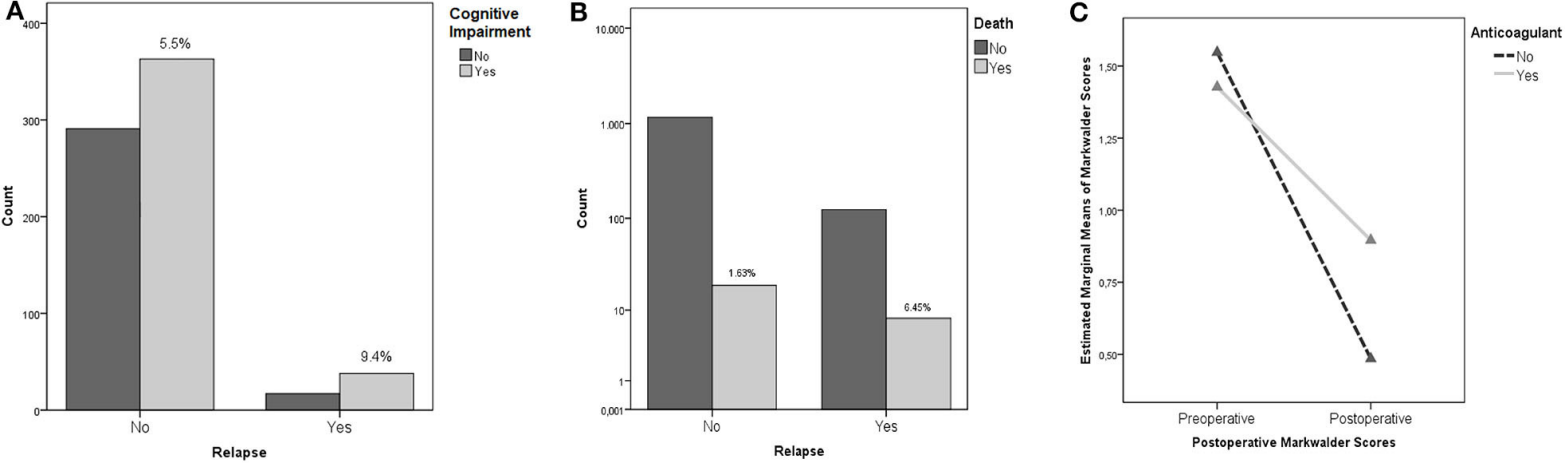
**(A)** Cognitive Impairment and risk of recurrence (the percentages concern the specific subgroup of patients who presented cognitive impairment in the preoperative period). **(B)** Recurrence and mortality. The percentages, namely the fatality rate, is significantly higher in patients who experienced a recurrence of CSDH. **(C)** ANOVA Repeated Measures analysis demonstrating the better outcomes (as measured with Markwalder scale) of patients with history of anticoagulant intake undergoing a postoperative corticosteroid therapy.

Neurological outcome was not associated with the rate of recurrence (*p* = 0.22) being a repeated surgery no factor predicting a worse outcome. The only clinical feature associated to the incidence of relapses was the cognitive slowing (9.4 vs. 5.5% - *p* = 0.034), being probably the clinical expression of a generalized cortical dysfunction, which was associated to a higher risk of relapses (Figure 2A); notably, the risk of fatality was higher in the subgroup of patients who presented a preoperative motor deficit (*p* = 0.026). For what our cohort is concerned, according to our analyses, relapse of a CSDH is, in our cohort, a statistically significant predictor of increased fatality risk (*p* = 0.004, OR 3.939 95% IC 1.689–9.184 – Figure 2B).

### Other Analyses

Multivariate ANOVA analyses were performed to rule out the possible influence of confounding factors. In particular, we found an interesting statistically significant interaction in patients under anticoagulant therapy with an history of trauma: presurgical corticosteroid treatment was associated to better postoperative functional results as measured with the Markwalder scale (*p* = 0.001). Moreover, presurgical corticosteroid therapy was associated to better Markwalder scores in patients who presented a history of trauma (*p* = 0.002). The better Markwalder outcomes, were, in our cohort, experienced by patients undergoing both a pre and postoperative corticosteroid therapy (*p* = 0.043). ANOVA Repeated Measures analyses investigated the endpoint variables: patients undergoing anticoagulant therapy, experienced averagely statistically significant worse outcomes as measured with Markwalder scores, in respect to patients whose therapy regime did not include anticoagulants, independently from their preoperative conditions (*p* = 0.027 – Figure 2C).

Logistic Regression Analyses were added in order to predict the postoperative functional status, concerning the preoperative use of antiplatelet and anticoagulant agents, pre and postoperative use of corticosteroid therapy, surgical drain, laterality of the hematoma and the impact on the functional results of operating a relapse. The results are summarized in Table 3. Notably, the number of patients who experience a postoperative improvement, in case of history of anticoagulant or antiplatelet agents intake ranges between 31.5 and 35.3% (*p* = 0.23), and concerning the corticosteroid therapy, a strong statistically significant (*p* = 0.036) prediction of 43.2% of patients experiencing an improvement of their conditions supports the postoperative use of corticosteroids.

**Table 3:** Multinomial logistic regression predicting the outcomes of patients on the ground of their antiplatelet/anticoagulant and corticosteroid treatments.

**Post-corticosteroid therapy**	**Pre-corticosteroid therapy**	**Postoperative status (Variation of Markwalder scores)**		**Frequency**			**Percentage**	
				**Observed**	**Predicted**	**Pearson residual**	**Observed**	**Predicted**
No	No	Worsened		4	3.934	0.033	1.0%	1.0%
		Stable		259	260.011	−0.108	66.2%	66.5%
		Improved		128	127.055	0.102	32.7%	32.5%
	Yes	Worsened		1	1.066	−0.065	4.2%	4.4%
		Stable		20	18.989	0.508	83.3%	79.1%
		Improved		3	3.945	−0.521	12.5%	16.4%
Yes	No	Worsened		2	2.066	−0.046	1.2%	1.2%
		Stable		95	93.989	0.157	56.2%	55.6%
		Improved		72	72.945	−0.147	42.6%	43.2%
	Yes	Worsened		7	6.934	0.026	5.8%	5.8%
		Stable		84	85.011	−0.203	70.0%	70.8%
		Improved		29	28.055	0.204	24.2%	23.4%
**Antiplatelet**	**Anticoagulant**	**Postoperative status (Variation of Markwalder scores)**		**Frequency**			**Percentage**	
				**Observed**	**Predicted**	**Pearson residual**	**Observed**	**Predicted**
No	No	Worsened		11	11.027	−0.008	2.6%	2.6%
		Stable		276	274.505	0.154	66.2%	65.8%
		Improved		130	131.469	−0.155	31.2%	31.5%
	Yes	Worsened		2	1.973	0.019	1.9%	1.9%
		Stable		67	68.495	−0.309	64.4%	65.9%
		Improved		35	33.531	0.308	33.7%	32.2%
Yes	No	Worsened		1	0.973	0.027	0.5%	0.5%
		Stable		117	118.495	−0.231	63.9%	64.8%
		Improved		65	63.531	0.228	35.5%	34.7%
	Yes	Worsened		0	0.027	−0.163	0%	0.4%
		Stable		6	4.505	1.180	85.7%	64.4%
		Improved		1	2.469	−1.162	14.3%	35.3%

## Discussion

The present multi-institutional analysis of 1,313 surgical cases of CSDH found then no association between the risk of recurrence and age, use of antithrombotic medication, or whether the hematoma was unilateral or bilateral. Surgical management influenced risk of recurrence, with patients receiving burr-hole craniostomy having the lowest recurrence rate, followed by patients undergoing a wider craniectomy and, lastly, patients receiving a craniotomy. Placement of a surgical drain was associated with a significantly reduced risk of recurrence. Finally, corticosteroid treatment with Dexamethasone was found to be associated with lower recurrence rates. Several papers regarding the treatment of CSDHs have recently been published; for the most part the results from the present study confirm previous findings reported in the literature, providing strengthened evidence in relation to the numerosity of the investigated population.

Chronic subdural hematoma is a common disorder that affects especially elderly people and is associated with substantial morbidity and mortality (5). In the general population its incidence is estimated to be between 1.72 and 20.6 per 100,000 per year, but it is higher for people aged 70 years and older (58 per 100,000 per year) (10–12). Because of the growing proportion of people aged 65 years and older, which is expected to double worldwide between 2000 and 2030 (13), a large rise in the incidence of CSDHs is expected and, consequentially, an increased interest toward the CSDH treatment has been highlighted in the last years (6). Moreover, small differences in surgical or medical treatment can potentially have a great impact on risk of recurrence, making optimization of clinical management of these patients a necessity with extensive repercussions.

Whether age and sex have an influence on the rates of recurrence after surgery for CSDH evacuation is debated, with some reports suggesting older age and male sex to be at higher risk (2) and others with opposite findings (14). In another series, a median age of 78 (interquartile range 70–84) for patients who did not have a recurrence has been highlighted, compared to a median age of 80 (76–87) for patients who did (15). Though such finding was statistically significant, it is arguable that a 2-year difference in age is not clinically relevant. Other authors pointed out in their series that average age was 74 for patients who did not need reoperation, vs. 74.5 for patients that required a second operation (14). This difference was reported not to be statistically significant; it is interesting to point out the difference in average age detailed in different series and different populations.

Use of antiplatelet or anticoagulant medication has consistently been found not to be significantly associated with an increased risk of CSDH recurrence (4, 16). In a Swedish study, preoperative antithrombotic treatment was found not to influence rate of recurrence at 3 months or perioperative mortality; it did, however, increase the risk of perioperative morbidity (17). Some authors have suggested that resumption of anticoagulant therapy is safe 72 h after surgery (4), while antiplatelets should be withheld for at least 7 days following CSDH drainage (7). Results of the present study support the idea that preoperative antithrombotic therapy does not increase the risk of recurrence of CSDH after evacuation; however, treatment should be tailored to each patient, taking specific cardiovascular risks into consideration.

Type of surgical evacuation for CSDHs has been a matter of debate. Though bedside twist-drill craniostomy has been successfully employed (18), a single or double burr-hole craniotomy under local or general anesthesia is usually the procedure of choice. Extended craniectomy or craniotomy that allow for resection of the capsule or membrane of the hematoma have been shown neither to provide advantages in lowering recurrence rates nor to improve the neurological outcome (19). The results of the present study showed a higher risk of recurrence when extended craniectomy or craniotomy was performed. However, this might depend on the specific characteristics of the hematomas that were chosen to be evacuated with these wider approaches, perhaps hematomas with a subacute component; in other words, these results might be distorted by selection bias. In this series the procedure of choice was single burr-hole craniostomy. It has been reported that a double burr-hole does not provide a significant clinical advantage and leads to higher rates of recurrence (20, 21). The rates of recurrence presented in our series suggest that the less invasive choice of a single burr-hole is adequate for obtaining satisfactory clinical outcomes.

Many studies demonstrated that an important modifiable risk factor for CSDH recurrence is postoperative drainage. Drain placement has been associated with lower recurrence (3.1–10.5% with drain vs. 17–33% without) (17, 22–25). A randomized controlled trial (RCT) reported a reduction of recurrence rates from 24 to 9.3% and no additional complication with the use of a subdural drain (11); the trial was even stopped early because of the clear results. An Indian prospective randomized trial showed similar complications and mortality, but fewer recurrences with drain placement (26 vs. 9%) (26). Other study groups and meta-analyses reached the same conclusions (27–31). An Indian RCT reported a lower rate of recurrence with a single burr-hole with drain placement vs. two burr-holes (1.4 vs. 15.7%) (20). The present study corroborates such literature, providing yet clearer evidence to support the use of postoperative drainage in all cases of surgical evacuation of CSDH.

The position of the drain (subdural vs. subperiosteal) does not appear to modify the outcome (32). Some authors suggested that a subperiosteal drainage could help reduce seizures and infection, avoiding direct contact with the hematoma membranes; in their study re-intervention rate for recurrence was 9.3% with drain placement (33). Although a drain in the subdural space would be the most intuitive solution to allow for complete evacuation of the hematoma, it is arguable that, once a communication between the subdural and subperiosteal spaces is made through a craniostomy, both spaces are suitable for drain placement. Indeed, a recently published RCT demonstrates the non-inferiority of subperiosteal placement in terms of recurrence rates, in the setting of reduced complication rates (18). It has been stated that, when placing a subdural drain, it should not be inserted for more than approximately 4 cm (1). The duration of drainage may matter, but evidence is lacking; a Chinese study reported 6.6% recurrence with drain placement, ranging from 16.3% with drain removal prior to 3 days to 1.3% if removed thereafter (3). However, these results must be interpreted cautiously, as they were obtained retrospectively and timing of removal was decided on an individual non-randomized basis. The present study suggests that 48 h of postoperative drainage is adequate to provide satisfactory outcomes, without significant complications.

The anti-inflammatory and anti-angiogenetic properties of Dexamethasone have repeatedly been shown to be beneficial in treating CSDH, when associated to surgical management (26, 34). In a meta-analysis the adjuvant use of Dexamethasone resulted in a lower recurrence rate if compared with non-Dexametasone therapy (RR, 0.54; 95% CI, 0.33–0.88; *p* = 0.01) (12). Moreover, no additional complications or morbidity appeared to be associated with corticosteroid treatment in this setting (15). The mechanism of action of corticosteroids in CSDH has been associated to the inhibition of the formation granulation tissue, responsible for the creation of a capsule surrounding the hematoma and containing numerous newly formed, permeable capillaries, which could be responsible for recurrent bleeding (22, 35–37). The results of the present study substantiate the hypothesis that Dexamethasone is beneficial as an adjunct treatment to surgery. The lowest of recurrence rates were associated with a combination of preoperative and postoperative administration of corticosteroid therapy; postoperative treatment alone was still associated with a significant benefit compared to no corticosteroid therapy.

In the literature, mortality during hospitalization for SCSH ranges from 5 to 13.3% (31, 38, 39). In the present study, perioperative mortality was 2.2% with a fatal outcome in 29 cases.

### Limitations and Generalizability

Presented results must be considered in the context of the limitations of this study. The lack of randomization in the design of the study inevitably hinders generalizability of the conclusions that can be drawn. Moreover, the focus on surgical cases only, might not provide a complete depiction of the pathology at hand. However, the large number of the population that was analyzed and the multicenter design allow for suggesting specific indications regarding management of CSDHs. Since small differences in surgical or medical treatment can potentially have a great impact on risk of recurrence, the homogeneous protocol followed by the two centers constitutes a valid support to this study. This study, therefore, substantiates, reassumes, and emphasizes key concepts of CSDHs treatment, in accordance with data reported in the literature.

## Conclusion

In conclusion, no association was found between the risk of recurrence of CSDH after surgical evacuation and age, use of antithrombotic medication, or laterality. Burr-hole craniostomy was found to be associated with the lowest recurrence rate, when compared to other surgical procedures. Placement of surgical drain and Dexamethasone therapy were significantly associated with reduced risk of recurrence of CSDHs.

## Data Availability Statement

The original contributions presented in the study are included in the article/supplementary material, further inquiries can be directed to the corresponding author.

## Ethics Statement

Ethical review and approval was not required for the study on human participants in accordance with the local legislation and institutional requirements. Written informed consent for participation was not required for this study in accordance with the national legislation and the institutional requirements.

## Author Contributions

FC: study concept, writing, and study analysis. AP: study concept, writing, and data analysis. GV: data analysis. MMa: writing. AM, MMi, GD'A, MP, and CF: data collection. ML, AF, and DG: supervision. FT and FZ: study analysis. All authors contributed to the article and approved the submitted version.

## Conflict of Interest

The authors declare that the research was conducted in the absence of any commercial or financial relationships that could be construed as a potential conflict of interest.
